# Biopsychosocial risk factors of depression during menopause transition in southeast China

**DOI:** 10.1186/s12905-022-01710-4

**Published:** 2022-07-05

**Authors:** Ketan Chu, Jing Shui, Linjuan Ma, Yizhou Huang, Fan Wu, Fang Wei, Xingjun Meng, Jie Luo, Fei Ruan, Jianhong Zhou

**Affiliations:** 1 School of Medicine, Women’s Hospital, Zhejiang University, 1st Xueshi Rd, Hangzhou, 310006 Zhejiang Province China; 2 Ninghai Maternal and Child Health Hospital, Ningbo, Zhejiang Province China; 3 Department of Pharmacy, The First Affiliated Hospital of Zhejiang Chinese Medical University, Hangzhou, 310006 Zhejiang Province China; 4 Department of Epidemiology and Biostatistics, Zhejiang University School of Medicine, Hangzhou, Zhejiang China

**Keywords:** Depression, Menopausal symptom, Menopause transition, Risk factors

## Abstract

**Objective:**

More than 2 billion women are experiencing menopause transition in China and some of them suffered from depression; while the risk factors of depression during menopause transition were still unclearin China. We aimed to investigate the risk factors in mid-life women in Southeast China.

**Method:**

This study included 1748 Chinese women aged 40–65 years-old who visited gynecology outpatient department of Women’s hospital School of Medicine, Zhejiang University during 2010–2018. Demographic information was collected, and the modified Kupperman Menopausal Index (mKMI) and Hamilton Rating Scale for Depression were assessed. Circulating levels of sex hormones were tested. Ordinal logistic regression analysis was performed to identify risk factors for depression.

**Results:**

The prevalence of depression symptoms was 47.43%. The majority of women had mild (38.56%) or moderate depressive symptoms (8.00%); only 0.86% had severe depressive symptoms. Compared with perimenopausal women, postmenopausal women had increased risks of more severe depression. The associations between menopausal syndromes and the intensity of depression were strongly positive (OR 6.69, 95% CI 5.39–8.29). Elder age, higher follicle stimulating hormone levels, lower estradiol levels, and fewer parity were positively related with the intensity of depression. Among postmenopausal women, underweight, mKMI > 14, earlier age at menopause, shorter reproductive period, and longer duration after menopause were risk factors for incresed intensity of depression.

**Conclusions:**

The results demonstrated a high proportion of depression in women complaining of menopause. Menopausal symptoms were strongly related to the intensity of depression. In postmenopausal women, estrogen related events are associated with the intensity of depression. Gynecological endocrinologists in China should consider screening for depression in high-risk women.

**Supplementary Information:**

The online version contains supplementary material available at 10.1186/s12905-022-01710-4.

## Introduction

In 2019, more than 95 million Chinese people sufffered from depression, 65% of them are female [1]. Worldwide epidemiological studies consistently showed that women had a two-times increased risk of depressive disorders compared with men. The risk was especially pronounced at life stages with sex hormones fluctuations, such as the postnatal period and the menopause transition [2]. Recent results of the Study of Women's Health across the Nation Mental Health Study (SWAN MHS) showed a three-fold increased risk of the development of major depression during the late perimenopausal or postmenopausal period compared with the pre- or early perimenopausal period [3]. China is an ageing scoiety, according to World Health Organization (WHO) statistics, there were 160 million postmenopausal women in China in 2010, and the number of postmenopausal women will reach 280 million by 2030. The incidence of depressive symptoms in women going through menopausal transition has increased significantly. Thus, recognizing depressive symptoms and disorders at an early stage is very important for the management of menopause transition.

Numerous studies have shown a wide range of risk factor domains for depressive symptoms and disorders during the menopause transition, including demographic factors (age, the body mass index (BMI), unemployment, financial problems), health factors (history of depressive disorders), psychosocial factors (upsetting life events,anxiety and depressive symptoms (for major depressive disorder)), hormonal levels (higher follicle stimulating hormone (FSH), luteinizing hormone (LH) and estradiol (E2) level) and menopausal symptoms (vasomotor symptoms, night sweats, increased number of bothersome symptoms) [4–6]. Recently, several studies were conducted to determine whether hormone-related events, such as duration of estrogen exposure years, reproductive events, and breast feeding history were related to the risk of depression during menopause transition [7, 8]. However, data on most of these associations are limited and controversial.

In the present study, we aimed to investigate the severity of depression among women experiencing menopause transition and analyze the relationships between demographic characteristics, menopausal symptoms, hormone levels, and depressive symptoms, and further reveal the related factors for depression. Our findings are expected to help health care providers all over the world, especially the gynecological endocrine doctors and general practitioners, to recognize and treat menopausal depressive disorders in early stage, which is very important to prevent severe depression in mid-life women.

## Methods

### Participants

Subjects were recruited from the gynecology outpatient department of the Women’s Hospital, School of Medicine, Zhejiang University between March 2010 and December 2018. Women who visited the outpatient clinic for the first time either because of complains about changes in their menstrual cycle or other symptoms related to menopause were asked to provide informed consent for the study. The following inclusion criteria were applied: (1) Aged between 40 and 65 years-old; (2) a perimenopausal or postmenopausal status. Women with any of the following conditions were excluded: (1) History of mental diseases or use of antipsychotic drugs; (2) History of sex steroids or oral contraceptives use within the preceding 6 months; (3) History of hysterectomy or oophorectomy; (4) History or evidence of uncontrolled hypertension, diabetes, cardiovascular disease, untreated thyroid disease, renal insufficiency, liver disease, life threatening disease, history of thrombosis, breast cancer, and inability to participate.

The ethical committee of the the Women’s Hospital, School of Medicine, Zhejiang University approved this study.

### Demographic data

Demographic and clinical data including age, residence, academic education, monthly income, parity, times of abortion, age at menarche, age at menopause (for postmenopausal women) and chronic health problem were collected by trained interviewers. The reproductive period was calculated as age at menopause minus the age at menarche; the duration after menopause was calculated as monthes between date at visit and the date at menopause. The body mass index (BMI, kg/m^2^) was calculated and classified according to the Chinese World Health Organization criteria: underweight, BMI < 18.5; normal, 18.5 ≤ BMI < 24; overweight, 24 ≤ BMI < 28; obesity, BMI ≥ 28.

### Assessment of the Hamilton Rating Scale for Depression (24-items) (HAM-D24)

The patients were assessed for depressive symptoms and severity using HAMD-24, which contains 24 items (10 items scored from 0 to 2, and 14 items scored from 0 to 4) A total score in range of 0–8, 9–19, 20–34, and ≥ 35 were considered as an indication of non-depression, mild, moderate, and severe depression, respectively.

### Assessment of menopausal symptoms and sex steroid concentrations

We made a diagnosis of perimenopausal or postmenopausal status according to the 2012 Stages of Reproductive Aging Workshop (STRAW) criteria [9].

Menopausal symptoms were assessed using the modified Kupperman Menopausal Index score (mKMI) [10].

For women with no menstruation, blood samples were collected for hormone tests. Women who still had an identifiable menstrual cycle were asked to undergo hormone tests on the second day of the cycle. The sample was collected from each subject the next morning between 8 and 11 am after overnight fast. Levels of E2, progesterone (P), testosterone (TE), LH and FSH were estimated by ELISA with Roche Modular E170 Analyser, Berlin, Germany. To analyze E2, there was a lower limit of detection of 18.35 pmol/L.

### Statistical analysis

Continuous variables with a normal distribution were presented as the mean ± standard deviation and we used one-way analysis of variance (ANOVA) for comparisons Categorical variables were presented as frequency and proportions and compared using chi-squared tests. Ordinal logistic regression analyses with the intensity of depression as the outcome were conducted. The estimates of logistic regression were presented as odds ratios (ORs) and 95% confidence intervals (CIs). In the categorical analyses for E2, for which there was a limitation of detection, women with E2 ≤ 18.35 pmol/L were categorized separately and the remaining women were divided into two groups by median values of E2 with the highest E2 group as the reference category. For categorical analyses of other sex steroids, participants were divided into four groups according to the quartile distribution of each steroid serum level with the lowest fourth as the reference category.

SPSS 23.0 for Windows (IBM Corp., Armonk, NY, USA) was used for all statistical analyses with *p* < 0.05 as statistically significant.

## Results

### General characteristics of the participants

A total of 2304 women meeting the inclusion criteria signed informed consent for the study. We then excluded those with history of sex steroids or oral contraceptives use within the preceding 6 months (n = 302), history of mental diseases or the use of antipsychotic drugs (n = 47), history of hysterectomy or oophorectomy (n = 103), history of hysterectomy or oophorectomy (n = 65) and those with uncontroled diseases (n = 39). Thus, a total of 1,748 women were recruited.

The general characteristics of participants are summarized in Table 1. The mean age of the 1,748 women were 48.56 ± 4.60 years old. Most of them were perimenopausal (66.36%), living in urban area (83.52%), employed (73.34%), and highly educated (32.84% with high school and 45.14% with college and above). The mean BMI was 22.19 ± 3.45 kg/m^2^, and 69.91% of them were considered as normal. Only 4.17% of the study group had chronic health problems. Age, age at menarche, personal income per month, employment status, and depression status were significantly different among women with different menopausal statuses (*p* < 0.05) (Table 1).

**Table 1:** General characteristics of participants

Variable	All	Menopausal status		Statistic	*p*
		Perimenopausal	Postmenopausal		
	n = 1748	n = 1160	n = 588		
Age (n, %)				*χ*^2^ = 336.434	< 0.001
40–49	1040 (59.50)	863 (74.40)	177 (30.10)		
50–59	685 (39.19)	297 (25.60)	388 (65.99)		
≥ 60	23 (1.32)	0	23 (3.91)		
Age (years, mean, SD)	48.56 ± 4.60	47.89 ± 6.34	51.65 ± 4.52	*t* = 22.74	< 0.001
Residence (n, %)				*χ*^2^ = 1.468	0.226
Urban	1460 (83, 52)	960 (82.76)	500 (85.03)		
Rural	288 (16.48)	200 (17.24)	88 (14.97)		
Education level (n, %)				*χ*^2^ = 111.532	< 0.001
Under high school	385 (22.02)	246 (21.21)	139 (23.64)		
High school	574 (32.84)	362 (31.21)	212 (36.05)		
College and above	789 (45.14)	552 (47.58)	237 (40.31)		
Employment status (n, %)				*χ*^2^ = 8.422	0.015
Employed	1282 (73.34)	943 (81.29)	339 (57.65)		
Unemployed or retired	466 (26.66)	217 (18.71)	249 (42.35)		
Personal income per month (yuan) (n, %)				*χ*^2^ = 11.180	0.004
< 2000	249 (14.25)	166 (14.31)	83 (14.12)		
2000–5000	791 (45,258)	494 (42.59)	297 (50.51)		
> 5000	708 (40.50)	500 (43.10)	208 (35.37)		
BMI (n, %)				*χ*^2^ = 3.079	0.38
Underweight (< 18.5 kg/m^2^)	102 (5.83)	74 (6.38)	28 (4.76)		
Normal (18.5–24 kg/m^2^)	1222 (69.91)	809 (69.74)	412 (70.07)		
Overweight (24–28 kg/m^2^)	282 (16.13)	189 (16.29)	94 (15.99)		
Obesity ( ≧ 28 kg/m^2^)	55 (3.15)	32 (2.76)	23 (3.91)		
Missing	87 (4.98)	56 (4.83)	31 (5.27)		
Past medical history (n, %)				*χ*^2^ = 0.636	0.752
High blood pressure	35 (2.00)	24 (2.07)	11 (1.87)		
Diabetes Mellitus	10 (0.57)	5 (0.43)	5 (0.85)		
Cancer	28 (1.60)	19 (1.64)	9 (1.53)		
Times of abortion (n, %)				*χ*^2^ = 0.657	0.714
N = 0	427 (24.43)	276 (23.79)	147 (25)		
1 ≤ N ≤ 2	992 (56.75)	666 (57.41)	326 (55.44)		
N ≥ 3	329 (18.82)	214 (18.45)	115 (19.56)		
Parity (n, %)				*χ*^2^ = 0.537	0.765
N = 0	154 (8.81)	105 (9.05)	49 (8.33)		
N = 1	1384 (79.18)	924 (79.66)	460 (78.23)		
N ≥ 2	210 (12.01)	131 (11.29)	79 (13.43)		
Age at menarche (years, mean, SD)	14.54 ± 1.58	14.46 ± 1.54	14.70 ± 1.65	*t* = 2.996	0.003
Age at menopause (years, mean, SD)			48.10 ± 3.82		
Reproductive period (years, mean, SD)			33.38 ± 3.77		
Duration after menopause (years, mean, SD)			3.62 ± 2.86		
Depression status (HAMD scores, n, %)				*χ*^2^ = 32.388	< 0.001
Non-depression (< 8)	919 (52.57)	653 (56.29)	266 (45.24)		
Mild depression (8–19)	674 (38.56)	428 (36.90)	246 (41.84)		
Moderate depression (20–34)	140 (8.00)	75 (6.47)	65 (11.05)		
Severe depression (≥ 35)	15 (0.86)	4 (0.34)	11 (1.87)		

### Assessment of depression

The mean HAMD score was 9.06 ± 7.44, and only 52.57% of the participants were considered as non-depressed (HAMD-24 less than 8). The majority of women with depression had mild (38.56%) or moderate symptoms (8.00%), only 0.86% had severe depression (Table 1). Age, employment status, menopausal status, personal income per month, and education were significantly different among women with different levels of depression (*p* < 0.05).

### Menopausal syndrome and depression

The mean mKMI score was 15.10 ± 9.24. The mean mKMI score of 919 women into non-depressiion group was 10.81 ± 6.42. The mean mKMI scores of participants with mild, moderate and severe depressive symptoms were 18.76 ± 8.11, 24.62 ± 11.17 and 35.53 ± 13.57, respectively. The incidence of the mKMI positives (mKMI > 14) was significantly different among the depression subgroups (*p* < 0.001). Significantly difference of mKMI scores was obervesed between the depression subgroups as well (Table 2).

**Table 2:** Prevalence of menopausal symptoms according to the modified Kupperman Menopausal Index by HAMD depression degrees

	Non-depression (< 8)		Mild depression (8–19)		Moderate depression (20–34)		Severe depression (≧35)		*p*^a^	*p*^b^
	n = 921		n = 672		n = 140		n = 15			
	Mean (SD)	Positive n (%)	Mean (SD)	Positive n (%)	Mean (SD)	Positive n (%)	Mean (SD)	Positive n (%)		
Hot flashes/sweating	3.35 (3.34)	546 (59.41)	4.72 (3.68)	494 (73.29)	5.62 (3.95)	111 (79.29)	6.67 (3.9)	13 (86.67)	< 0.001	< 0.001
Insomnia	1.3 (1.36)	504 (54.84)	2.38 (1.69)	546 (81.01)	3.05 (1.81)	126 (90.00)	4.13 (2.2)	13 (86.67)	< 0.001	< 0.001
Mood swings	1.25 (1.39)	476 (51.80)	2.28 (1.62)	538 (79.82)	2.97 (1.88)	117 (83.57)	4.53 (2.07)	14 (93.33)	< 0.001	< 0.001
Melancholia	0.25 (0.49)	201 (21.87)	0.74 (0.74)	384 (56.97)	1.32 (0.92)	111 (79.29)	2.07 (1.03)	13 (86.67)	< 0.001	< 0.001
Sexual problems	1.46 (1.45)	550 (59.85)	2.22 (1.65)	539 (79.97)	2.62 (1.89)	117 (83.57)	3.6 (2.53)	12 (80.00)	< 0.001	< 0.001
Muscle/joint pain	0.58 (0.69)	432 (47.01)	1.00 (0.83)	464 (68.84)	1.06 (0.93)	97 (69.29)	1.27 (1.22)	9 (60.00)	< 0.001	< 0.001
Vertigo	0.32 (0.52)	273 (29.71)	0.67 (0.71)	366 (54.30)	0.94 (0.91)	92 (65.71)	1.53 (1.25)	11 (73.33)	< 0.001	< 0.001
Fatigue	0.56 (0.6)	462 (50.27)	1.04 (0.68)	552 (80.90)	1.28 (0.9)	116 (82.86)	1.93 (1.16)	13 (86.67)	< 0.001	< 0.001
Headaches	0.34 (0.54)	285 (31.01)	0.62 (0.71)	340 (50.45)	0.85 (0.83)	88 (62.86)	1.33 (1.23)	10 (66.67)	< 0.001	< 0.001
Formication	0.27 (0.78)	112 (12.19)	0.59 (1.17)	159 (23.59)	1.2 (1.58)	63 (45.00)	1.73 (2.12)	8 (53.33)	< 0.001	< 0.001
Urinary tract infection	0.43 (1.07)	154 (16.57)	0.88 (1.41)	230 (34.12)	1.08 (1.66)	53 (37.86)	2 (2.39)	8 (53.33)	< 0.001	< 0.001
Palpitations	0.37 (0.58)	305 (33.19)	0.75 (0.74)	402 (59.64)	0.97 (0.8)	102 (72.86)	1.67 (0.9)	14 (93.33)	< 0.001	< 0.001
Paresthesia	0.33 (0.93)	117 (12.73)	0.96 (1.45)	231 (34.27)	1.66 (1.69)	79 (56.43)	3.07 (1.83)	12 (80.00)	< 0.001	< 0.001
mKMI total score	10.81 (6.42)	877 (95.43)	18.78 (8.11)	667 (98.96)	24.62 (11.17)	133 (95.00)	35.53 (13.57)	15 (100)	< 0.001	< 0.001

^a^*p*-value for comparison of severity of menopausal syndromes in each depression subgroup

^b^*p*-value for comparison of prevalence of menopausal syndromes in each depression subgroup

### Sex steroids and depression

Table 3 showed the E2 and FSH serum levels according to the categorical distribution of depression The mean value of serum E2 was 82.45 ± 137.97 (pmol/L). The population was divided into three groups as T1 (E2 ≤ 18.35), T2 (18.35 < E2 ≤ 62.16), T3 (E2 > 62.16). A significant decrease of depression severity was observed with increasing level of serum estradiol (Table 3).

**Table 3:** Estradiol and FSH serum levels according to the categorical distribution and depression cross-tabulation

	Non-depression (< 8)	Mild depression (8–19)	Moderate depression (20–34)	Severe depression (≥ 35)	*p*
E2 (pmol/L) (N, %)					< 0.001
T1 (E2 ≤ 18.35)	351 (40.91)	263 (42.15)	69 (53.08)	10 (83.3)	
T2 (18.35 < E2 ≤ 62.16)	237 (27.62)	193 (30.93)	38 (29.23)	2 (16.7)	
T3 (E2 > 62.16)	277 (32.28)	168 (26.92)	23 (17.69)	0 (0)	
Total	858	624	130	12	
FSH (IU/L) (N, %)					0.059
Q1 (FSH < 40)	140 (16.28)	70 (11.11)	13 (10.0)	0 (0)	
Q2 (40 ≤ FSH < 80)	418 (48.60)	326 (51.75)	62 (47.692)	5 (41.67)	
Q3 (80 ≤ FSH < 120)	251 (29.19)	188 (29.84)	48 (36.924)	6 (50.00)	
Q4 (FSH ≥ 120)	51 (5.93)	46 (7.30)	7 (5.39)	1 (8.33)	
Total	860	630	130	12	

The mean value of serum FSH level was 72.53 ± 35.63 (IU/L). The population was divided into four groups according to the FSH serum levels as Q1 (0 < FSH ≤ 40), Q2 (40 < FSH ≤ 80), Q3 (80 < FSH ≤ 120), Q4 (FSH > 120). No significant difference in the severity of depression was found in each FSH category (Table 3).

No significant associations were observed between depression and P(*p* = 0.359), TE(*p* = 0.404), and LH(*p* = 0.625) levels.

### Factors associated with the intensity of depression

Ordinal logistic regression analyses were performed to investigate independent factors associated with the intensity of depression (Table 4, Additional file 1: Table S1).

**Table 4:** Multivariable logistic regression analyses for factors associated with depression according to the HAMD

Population	Risk factor	OR	95% CI	*p*	Reference group
Total	Age^a^	1.04	1.01–1.06	0.004	*
	Underweight^a^	1.51	1.01–2.25	0.042	Normal BMI
	Postmenopause^a^	1.34	1.07–1.69	0.012	Perimenopause
	Parity (0)^a^	1.47	1.01–2.13	0.044	Parity (1)
	Parity (≥ 2)^a^	0.72	0.53–0.97	0.033	Parity (1)
	mKMI > 14	6.69	5.39–8.29	< 0.001	mKMI ≤ 14
	E2 T1	1.35	1.06–1.73	0.016	E2 T3
	E2 T2	1.36	1.07–1.77	0.012	E2 T3
	FSH Q4	1.62	1.00–2.62	0.048	FSH Q1
	FSH Q3	1.6	1.15–2.23	0.005	FSH Q1
	FSH Q2	1.44	1.06–1.97	0.022	FSH Q1
Postmenopausal	Underweight	2.22	1.06–4.63	0.034	Normal BMI
	mKMI > 14	6.18	4.25–8.98	< 0.001	mKMI ≤ 14
	Age at menopause	0.93	0.87–0.99	0.033	*
	Reproductive period	0.93	0.87–1.00	0.041	*
	Duration after menopause	1.08	1.01–1.16	0.03	*
Perimenopausal	Age	1.04	1.00–1.07	0.041	*
	Residence in rural area	1.45	1.03–2.06	0.035	Residence in urban area
	Parity (0)	1.93	1.22–3.05	0.005	Parity (1)
	Parity (≥ 2)	0.67	0.45–1.00	0.048	Parity (1)
	mKMI > 14	7.22	5.52–9.45	< 0.001	mKMI ≤ 14

Values are presented as OR (95% CI). Adjusted for Age, place of residence, level of education, employment, income, parity, times of abortion, age at menarche and BMI

^a^Values are presented as OR (95% CI).Adjusted for Age, place of residence, level of education, employment status, income, parity, times of abortion, age at menarche and BMI

CI, confidence interval; OR, odds ratio

*Variables was anaylsis as continuous variable

Age, menopausal status, parity, mKMI score, serum E2, and FSH levels were significantly correlated with the intensity of depression. Postmenopausal stages was positively correlated with the intensity of depression (OR 1.34, 95% CI 1.07–1.69). Women who had never given birth to a child had an increased risk of more severe depression (OR 1.47, 95% CI 1.01–2.13), while more than two parities was negatively correlated with the intensity of depression compared with women who had only one parity (OR 0.72, 95% CI 0.53–0.97). A nearly six times increased risk was observed in women with mKMI score more than 14 (OR 6.69, 95% CI 5.39–8.29).

A significant negative association was found between circulating E2 levels and the intensity of depression. The severity of depression decreased in participants with higher levels of estradiol (T1:T3 OR 1.35, 95%CI 1.06–1.73; T2:T3 1.36, 95%CI 1.04–1.78). For FSH analysis, the opposite result was observed, serum FSH level was positively related to the severity of depression (Q4:Q1 OR 1.62, 95%CI 1.00–2.62; Q3:Q1 OR 1.60, 95%CI 1.15–2.23; Q2:Q1 OR 1.44, 95%CI 1.06–1.97).

For postmenopausal women, we also collected information about the reproductive period, age at menopause, and duration after menopause. The results supported protective effect of later menopause (OR 0.93, 95% CI 0.87–0.99), longer reproductive period (OR 0.93, 95% CI 0.87–1.00), and a potential harmful effect of longer duration after menopause (OR 1.08, 95% CI 1.01–1.16) on the intensity of depression after menopause. Consistent with the whole population, underweight (OR 2.19, 95% CI 1.04–-4.57) and mKMI > 14 (OR 6.18, 95% CI 4.25–-8.98) were also potential risk factors of incresed intensity of depression in postmenopausal women.

In perimenopausal women, age, residence in rural area, parity, and mKMI score were significantly positively related to with the intensity of depression and an mKMI > 14 (OR 7.22, 95% CI 5.52–-9.45) was strongly associated with the intensity of depression as well.

The analyses showed storng associations between mKMI score and the severity of depression. Concerning there were some overlaping symptoms between mKMI and HAMD scales, we analyzed each menopausal symptoms in the mKMI scale sepreately. We found all the 13 symptoms were significantly related to with the severity of depression. Except the melancholia, the most related symptoms were fatigue (OR 3.09, 95% CI 2.68–3.56), vertigo (OR 2.62, 95% CI 2.27–3.02), palpitation (OR 2.51, 95% CI 2.19–2.88) and headache (OR 2.19, 95% CI 1.90–4.52) (Additional file 2: Table S2). The subgroup analysis dividing the time interval into four durations showed our results were stable (Additional file 3: Table S3).

## Discussion

In the present study, we explored the risk factors associated with depression among women who had menopausal symptoms in southeast China. We found that nearly half of the participants who visited the gynecology clinic (47.43%) had depression. The majority of women with depression had mild (38.56%) or moderate symptoms (8.00%), and only 0.86% had severe depression. Compared with perimenopausal women, postmenopausal women had increased risks of more severe depression. The relationships between menopausal syndromes and the intensity of depression were strong and positive. Women with elder age, higher serum FSH levels, lower serum E2 levels, and fewer parity had an increased risk of more severe depression.

In this hospital-based study, a relatively higher prevalence of depression (47.75%) was found in middle-aged women compared with other studies conducted in China (11.4–36.3%) [1, 6, 11, 12]. This might be explained by the fact that most of our participants had complaints about menopause, which may lead to increased the risk of depression in this population [12]. Our findings suggested a positive relationship between age and depression during menopausal transition, which was consistent with previous studies in China [1, 13]. Several studies stated that poor general health status of older women might contribute to this relationship [1]. However, in our study, most of the participants had good health status (95.83% did not have disease history), which suggested that the higher prevalence of depression in older women was mainly caused by the physiological and psychological changes during menopause transition.

In our study, we found strong positive correlations between menopausal symptoms (measured by mKMI) and the intensity of depression. Certain menopausal complaints co-occurred and overlapped with the presentation of mood disturbance during this stage. The associations between menopausal symptoms and depression had been reported by several studies [11, 12, 14, 15]. However, most studies focused on vasomotor symptoms (VMS), sleeping difficulties or other symptoms separately [12, 14]. Our result showed that not only VMS and sleeping difficulties, but also the other menopausal symptoms are associated with incresed intensity of depression in this period, and women with elevated mKMI scores was strongly associated with the intensity of depression during the menopause transition and the most related symptoms are fatigue, vertigo, palpitations and headaches.

The association between menopause status and the intensity of depression has been studied extensively [16, 17], positive correlation between perimenopause and depression has been observed in both cross-sectional studies and longitudinal studies. For example, a meta-analysis reported that the OR of the presence of depressive symptoms during the perimenopause were doubled when compared with those for premenopausal women. However, the analysis comparing the occurrence of depressive symptoms in the perimenopause versus that in the postmenopause revealed negative results [17]. By contrast, our results showed positive correlation with postmenopausal state and the intensity of depression. A possible explanation for the inconsistent results is that most of the postmenopausal women in our study were in the early postmenopausal stage and had complains of menopausal symptoms; thus, these women experienced a longer period with unpleasant feelings, which may lead to more depression symptoms.

The relationship between body mass index (BMI) and depression during the menopause transition is controversial. Several studies indicated associations between overweight, obesity and depression [18, 19], while others did not [11]. Studies of BMI and depression in general Asian populations had reported a U-shaped pattern of relationship with BMI categories [20]. Our study also indicated a higher risk of incresed intensity of depression in underweight women compared with women withnormal BMI. However, we did not find a significant correlation between overweight, obesity and the intensity of depression, which could be explained by the fact that our population was generally healthy, and chronic diseases were rare, even in the obesity women.

Estrogen exposure may influence women's risk of depression [21]; therefore, we analyzed the effect of hormone-related events on the risk of incresed intensity of depression. The majority of our participants were perimenopausal women who still underwent menstruation; therefore, we collected data concerning parity, times of abortion, and age at menarche in the whole population. The results only supported the protective effect of more parity on the risk of incresed intensity of depression during the menopause transition, which was consistent with the results from some previous studies [22, 23].

In postmenopausal women in whom menstrual events reflected endogenous hormone exposure, clinical and epidemiologic data supported a protective role for a longer estrogen exposure period in mood disorders [21, 24]. Consistent with these studies we observed a protective effect of longer reproductive period and later age at menopause on the risk of incresed intensity of depression after menopause. Interestingly, in our study, longer duration after menopause was found to have harmful effect after adjusting for demographic variables. Previous studies examining this association in postmenopausal women were scarce, only one American cohort study recruiting 203 women evaluated the depressive symptoms around natural menopause and indicated a decrease of approximately 15% of baseline per year of the CES-D score [25]. As in our study, the negative association of duration after menopause and the intensity of depression might be an indirect indication of the early use of HRT after menopause to prevent depression, which was proven by a recent clinical trial [26]. However, because of the limited evidence, further studies should be conducted to discuss the development of depression after menopause.

Numerous studies on animals and cells demonstrated a beneficial effect of estrogen on mood. Epidemiological studies found heterogeneous results, lower serum levels of E2 [28, 29] and higher levels of FSH [5, 27] have been associated with depressive symptoms in perimenopausal and postmenopausal women. However, other studies found no relation between estrogen or FSH levels and mood or depression [29, 30]. Meanwhile, some investigators identified that fluctuations in estrogen levels over time were more closely linked to depression than absolute hormone levels [31, 32]. Our results suggested women with higher level of serum E2 and lower serum FSH had less intensity of depression. Together with our findings of the associations between depression and hormone-related events, our results supported the view that more estrogen exposure decreased the risk of incresed intensity of depression in midlife women in China.

This study has several strengths. First, our study is one of the few studies to show factors related to the intensity of depression during the menopausal transition in southeast China. Second, this is the first study to comprehensively measure the demographic, symptomatic, and hormonal risk factors of the the intensity of depression, as well as historical events in Chinese population. Third, some important new findings were revealed, including the association between the mKMI score, duration after menopause, and the risk of incresed intensity of depression. Further, our study showed a relatively high prevalence of depression in women who had complaints about menopause; therefore, gynecologists as the first health care providers for these women not only in China,but also in the whole world should not only focus on the treatment of menopausal symptoms, but also concerned about the mental state of the patients. Thus screening for depression during consultations in mid-life women with menopausal symptoms should be considered for the early detection and prevention of severe depressive disorders.

While, there are several limitations of the present study. First, our findings were based on a cross-sectional study; therefore, we could only report the potential risk factors of depression and causal inferences could not be made. The time interval of the study was relatively long, thus, we did the subgroup analysis dividing the time interval into four durations, the result showed our results were stable. Second, some the data were collected by self-reporting. This could lead to inaccurate information such as childbearing history and menstrual history, which occurred many decades before. However, a high degree of accuracy has been reported for women recalling their reproductive events [33]. Third, our population was hospital based, most of the patients came to us because of menopausal symptoms, our population could not represent all the women experiencing menopausal transition, which might cause selection bias. Further population-based studies and meta-analyses are expected to minimize bias and systematic error.

## Conclusions

The present study showed elder age, underweight, postmenopause, lower serum level of E2, and higher serum level of FSH were positively associated with the intensity of depression, and increased mKMI score was strongly related with the intensity of depression. Increased number of parities was negatively associated with the intensity of depression in Chinese women during menopausal transition.

Sometimes, depression during menopause transition may be hard to be identified, The results of the present study might be helpful to improve the understandings of the associated factors with depression during menopause transition and to improve clinical management and public health programs to prevent and detect depression in the very early phase in high-risk mid-life women.

## Supplementary Information

**Additional file 1**. Table of multivariable logistic regression analyses for factors not meaningful.

**Additional file 2**. Table of multivariable logistic regression analyses for menopausal symptoms with depression according to the HAMD.

**Additional file 3**. Subgroup analysis of factors associated with depression according to the HAMD in each time intervals.

## Data Availability

The datasets used and/or analysed during the current study are available from the corresponding author on reasonable request.

## Abbreviations

mKMI: Kupperman Menopausal Index
HAMD-24: 24-Item Hamilton Rating Scale for Depression
WHO: World Health Organization
SWAN MHS: Study of Women's Health across the Nation Mental Health Study
E2: Estradiol
P: Progesterone
TE: Testosterone
LH: Luteinizing hormone
FSH: Follicle stimulating hormone
STRAW: 2012 Stages of Reproductive Aging Workshop
ANOVA: One-way analysis of variance
OR: Odds ratio
CI: Confidence interval
