# Supplementary material for: Biopsychosocial risk factors of depression during menopause transition in southeast China
Source: BMC Womens Health. 2022 Jul 5;22:273. doi: 10.1186/s12905-022-01710-4 (PMC9258098; doi:10.1186/s12905-022-01710-4)
Supplement: Supplementary file 1 — Additional file 1. Table of multivariable logistic regression analyses for factors not meaningful. [file 12905_2022_1710_MOESM1_ESM.docx]

| Supplementary table 1. Multivariable logistic regression analyses for factors not meaningful | | | | | |
| --- | --- | --- | --- | --- | --- |
| Population | Risk factor | OR | 95% CI | *p* | Reference group |
| Total | Overweight (24~28 kg/m2)^a^ | 0.87 | 0.67-1.13 | 0.283 | Normal BMI |
|  | Obesity ( ≧ 28 kg/m2)^a^ | 0.99 | 0.58-1.69 | 0.977 | Normal BMI |
|  | Residence in rural area^a^ | 1.32 | 0.99-1.75 | 0.057 | Residence in urban area |
|  | Unemployment^a^ | 1.15 | 0.89-1.48 | 0.293 | Employment |
|  | Education(Under high school)^a^ | 1.28 | 0.93-1.78 | 0.131 | Education(College and above) |
|  | Education(High school)^a^ | 1.14 | 0.89-1.46 | 0.319 | Education(College and above) |
|  | Income(<2000Yuan/Month) | 1.43 | 0.99-2.05 | 0.056 | Income(>5000Yuan/Month) |
|  | Income(2000-5000Yuan/Month) | 1.12 | 0.88-1.42 | 0.365 | Income(>5000Yuan/Month) |
|  | Age at menarche^a^ | 0.98 | 0.91-1.04 | 0.435 | * |
|  | Times of abortion(N=0)^a^ | 0.98 | 0.76-1.25 | 0.857 | Times of abortion(1≤N≤2) |
|  | Times of abortion(N≥3)^a^ | 1.21 | 0.94-1.55 | 0.135 | Times of abortion(1≤N≤2) |
|  | P Q1 | 0.85 | 0.61-1.16 | 0.304 | P Q4 |
|  | P Q2 | 0.89 | 0.65-1.22 | 0.46 | P Q4 |
|  | P Q3 | 0.82 | 0.59-1.12 | 0.204 | P Q4 |
|  | LH Q4 | 1.26 | 0.95-1.67 | 0.105 | LH Q1 |
|  | LH Q3 | 1.26 | 0.95-1.67 | 0.103 | LH Q1 |
|  | LH Q2 | 1.07 | 0.81-1.42 | 0.625 | LH Q1 |
|  | T Q1 | 0.84 | 0.61-1.14 | 0.262 | T Q4 |
|  | T Q2 | 1 | 0.72-1.39 | 0.998 | T Q4 |
|  | T Q3 | 1.09 | 0.78-1.51 | 0.186 | T Q4 |
| Postmenopausal | Age | 1.04 | 1.00-1.09 | 0.052 | * |
|  | Overweight (24~28 kg/m2) | 0.87 | 0.67-1.13 | 0.585 | Normal BMI |
|  | Obesity ( ≧ 28 kg/m2) | 0.99 | 0.58-1.69 | 0.208 | Normal BMI |
|  | Residence in rural area | 1.03 | 0.62-1.72 | 0.912 | Residence in urban area |
|  | Unemployment | 1.06 | 0.71-1.57 | 0.792 | Employment |
|  | Education(Under high school) | 1.48 | 0.86-2.54 | 0.153 | Education(College and above) |
|  | Education(High school) | 1.47 | 0.97-2.23 | 0.073 | Education(College and above) |
|  | Income(<2000Yuan/Month) | 1.74 | 0.91-3.35 | 0.095 | Income(>5000Yuan/Month) |
|  | Income(2000-5000Yuan/Month) | 0.98 | 0.65-1.49 | 0.936 | Income(>5000Yuan/Month) |
|  | Age at menarche | 0.99 | 0.89-1.11 | 0.874 | * |
|  | Times of abortion(N=0) | 0.94 | 0.62-1.43 | 0.783 | Times of abortion(1≤N≤2) |
|  | Times of abortion(N≥3) | 1.18 | 0.78-1.78 | 0.442 | Times of abortion(1≤N≤2) |
|  | Parity(0) | 0.82 | 0.43-1.59 | 0.562 | Parity(N=1) |
| Perimenopausal | Parity(≥2) | 0.77 | 0.46-1.27 | 0.304 | Parity(N=1) |
|  | Underweight | 1.27 | 0.79-2.06 | 0.326 | Normal BMI |
|  | Overweight (24~28 kg/m2) | 0.78 | 0.56-1.07 | 0.126 | Normal BMI |
|  | Obesity ( ≧ 28 kg/m2) | 1.27 | 0.79-2.06 | 0.339 | Normal BMI |
|  | Unemployment | 1.21 | 0.86-1.71 | 0.28 | Employment |
|  | Education(Under high school) | 1.17 | 0.77-1.76 | 0.462 | Education(College and above) |
|  | Education(High school) | 0.99 | 0.72-1.35 | 0.914 | Education(College and above) |
|  | Income(<2000Yuan/Month) | 1.37 | 0.88-2.14 | 0.162 | Income(>5000Yuan/Month) |
|  | Income(2000-5000Yuan/Month) | 1.24 | 0.92-1.67 | 0.151 | Income(>5000Yuan/Month) |
|  | Age at menarche | 0.96 | 0.88-1.04 | 0.277 | * |
|  | Times of abortion(N=0) | 1.01 | 0.74-1.38 | 0.947 | Times of abortion(1≤N≤2) |
|  | Times of abortion(N≥3) | 1.22 | 0.89-1.67 | 0.22 | Times of abortion(1≤N≤2) |
|  | E2 T1 | 1.22 | 0.91-1.63 | 0.184 | E2 T3 |
|  | E2 T2 | 1.35 | 0.99-1.84 | 0.059 | E2 T3 |
|  | P Q1 | 0.84 | 0.56-1.24 | 0.376 | P Q4 |
|  | P Q2 | 0.98 | 0.66-1.45 | 0.909 | P Q4 |
|  | P Q3 | 0.76 | 0.51-1.14 | 0.186 | P Q4 |
|  | FSH Q4 | 1.29 | 0.91-1.81 | 0.15 | FSH Q1 |
|  | FSH Q3 | 1.36 | 0.97-1.91 | 0.079 | FSH Q1 |
|  | FSH Q2 | 1.05 | 0.75-1.48 | 0.774 | FSH Q1 |
|  | LH Q4 | 1.22 | 0.86-1.71 | 0.262 | LH Q1 |
|  | LH Q3 | 1.18 | 0.83-1.67 | 0.365 | LH Q1 |
|  | LH Q2 | 0.99 | 0.69-1.41 | 0.93 | LH Q1 |
|  | T Q1 | 0.72 | 0.48-1.06 | 0.097 | T Q4 |
|  | T Q2 | 0.8 | 0.53-1.20 | 0.281 | T Q4 |
|  | T Q3 | 0.88 | 0.53-1.33 | 0.538 | T Q4 |

Values are presented as OR (95% CI).Adjusted for Age, place of residence, level of education, employment,income, parity, times of abortion, age at menarche and BMI.

^a^ Values are presented as OR (95% CI).Adjusted for Age, place of residence, level of education, employment status, income, parity, times of abortion, age at menarche and BMI.

CI=confidence interval, OR=odds ratio.

*variables was anaylsis as continuous variable
