# Supplementary material for: Biopsychosocial risk factors of depression during menopause transition in southeast China
Source: BMC Womens Health. 2022 Jul 5;22:273. doi: 10.1186/s12905-022-01710-4 (PMC9258098; doi:10.1186/s12905-022-01710-4)
Supplement: Supplementary file 2 — Additional file 2. Table of multivariable logistic regression analyses for menopausal symptoms with depression according to the HAMD. [file 12905_2022_1710_MOESM2_ESM.docx]

Supplementary table 2. Multivariable logistic regression analyses for menopausal symptoms with depression according to the HAMD

|  | Total | | | Perimenopausal | | | postmenopausal | | |
| --- | --- | --- | --- | --- | --- | --- | --- | --- | --- |
| Risk factor | OR | 95% CI | *p* | OR | 95% CI | *p* | OR | 95% CI | *p* |
| Hot flashes/sweating | 1.13 | 1.10-1.15 | <0.001 | 1.15 | 1.10-1.20 | <0.001 | 1.15 | 1.10-1.18 | <0.001 |
| Insomnia | 1.59 | 1.49-1.69 | <0.001 | 1.72 | 1.55-1.92 | <0.001 | 1.72 | 1.55-1.92 | <0.001 |
| Mood swings | 1.62 | 1.53-1.72 | <0.001 | 1.66 | 1.50-1.83 | <0.001 | 1.66 | 1.50-1.83 | <0.001 |
| Melancholia | 4.02 | 3.49-4.65 | <0.001 | 3.94 | 3.13-4.97 | <0.001 | 3.94 | 3.13-4.97 | <0.001 |
| Sexual problems | 1.37 | 1.29-1.46 | <0.001 | 1.22 | 1.12-1.34 | <0.001 | 1.22 | 1.12-1.34 | <0.001 |
| Muscle/joint pain | 1.90 | 1.68-2.14 | <0.001 | 1.70 | 1.39-2.10 | <0.001 | 1.70 | 1.39-2.09 | <0.001 |
| Vertigo | 2.62 | 2.27-3.02 | <0.001 | 2.31 | 1.84-2.90 | <0.001 | 2.31 | 1.84-2.90 | <0.001 |
| Fatigue | 3.09 | 2.68-3.56 | <0.001 | 2.89 | 2.30-3.64 | <0.001 | 2.89 | 2.30-3.64 | <0.001 |
| Headaches | 2.19 | 1.90-4.52 | <0.001 | 2.12 | 1.68-2.68 | <0.001 | 2.12 | 1.68-2.68 | <0.001 |
| Formication | 1.58 | 1.45-1.72 | <0.001 | 1.48 | 1.28-1.71 | <0.001 | 1.48 | 1.28-1.71 | <0.001 |
| Urinary tract infection | 1.32 | 1.23-1.42 | <0.001 | 1.21 | 1.09-1.34 | <0.001 | 1.21 | 1.09-1.34 | <0.001 |
| Palpitations | 2.51 | 2.19-2.88 | <0.001 | 2.06 | 1.67-2.55 | <0.001 | 2.06 | 1.67-2.55 | <0.001 |
| Paresthesia | 1.66 | 1.54-1.78 | <0.001 | 1.64 | 1.45-1.84 | <0.001 | 1.64 | 1.45-1.84 | <0.001 |

Values are presented as OR (95% CI).Adjusted for Age, place of residence, level of education, employment, income, parity, times of abortion, age at menarche and BMI.

CI=confidence interval, OR=odds ratio.
