# Supplementary material for: Biopsychosocial risk factors of depression during menopause transition in southeast China
Source: BMC Womens Health. 2022 Jul 5;22:273. doi: 10.1186/s12905-022-01710-4 (PMC9258098; doi:10.1186/s12905-022-01710-4)
Supplement: Supplementary file 3 — Additional file 3. Subgroup analysis of factors associated with depression according to the HAMD in each time intervals. [file 12905_2022_1710_MOESM3_ESM.docx]

| Supplementary table 3. Subgroup analysis of factors associated with depression according to the HAMD in each time intervals | | | | | | | | | | | | | |
| --- | --- | --- | --- | --- | --- | --- | --- | --- | --- | --- | --- | --- | --- |
|  | Group1(2010.3-2012.12) | | | Group2(2013.1-2014.12) | | | Group3(2015.1-2016.12) | | | Group4(2017.1-2018.12) | | | Reference group |
|  | n=554 | | | n=340 | | | n=437 | | | n=412 | | |  |
| Risk factor | OR | 95% CI | *p* | OR | 95% CI | *p* | OR | 95% CI | *p* | OR | 95% CI | *p* |  |
| Age^a^ | 1.03 | 0.98-1.08 | 0.231 | 0.99 | 0.93-1.06 | 0.797 | 1.07 | 1.01-1.13 | 0.016 | 1.06 | 1.01-1.20 | 0.03 | * |
| Underweight^a^ | 2.15 | 1.06-4.34 | 0.033 | 1.12 | 0.39-3.19 | 0.833 | 2.22 | 1.02-4.81 | 0.044 | 0.96 | 0.41-2.25 | 0,932 | Normal BMI |
| Overweight (24~28 kg/m2)^a^ | 0.79 | 0.48-1.29 | 0.345 | 0.77 | 0.41-1.47 | 0.433 | 0.88 | 0.53-1.46 | 0.63 | 1.02 | 0.60-1.73 | 0.944 | Normal BMI |
| Obesity ( ≧ 28 kg/m2)^a^ | 1.1 | 0.40-3.05 | 0.851 | 0.98 | 0.32-3.01 | 0.964 | 0.7 | 0.20-2.43 | 0.576 | 1.59 | 0.57-4.34 | 0,376 | Normal BMI |
| Postmenopause^a^ | 1.36 | 0.89-2.07 | 0.154 | 1.19 | 0.70-2.03 | 0.524 | 1.61 | 0.99-2.62 | 0.057 | 1.18 | 0.75-1.87 | 0.476 | Perimenopause |
| Residence in rural area^a^ | 1.29 | 0.74-2.25 | 0.364 | 1.24 | 0.58-2.66 | 0.573 | 1.53 | 0.89-2.65 | 0.129 | 1.25 | 0.73-2.12 | 0.416 | Residence in urban area |
| Unemployment^a^ | 0.92 | 0.56-1.51 | 0.745 | 1.61 | 0.87-3.01 | 0.129 | 1.3 | 0.79-2.14 | 0.308 | 1.13 | 0.68-1.89 | 0.631 | Employment |
| Education(Under high school)^a^ | 1.69 | 0.93-1.78 | 0.103 | 1.2 | 0.52-2.73 | 0.672 | 1.09 | 0.57-2.09 | 0.803 | 1.34 | 0.70-2.55 | 0.38 | Education(College and above) |
| Education(High school)^a^ | 1.31 | 0.90-3.16 | 0.279 | 0.96 | 0.57-1.63 | 0.876 | 0.94 | 0.57-1.54 | 0.796 | 1.44 | 0.84-2.46 | 0.184 | Education(College and above) |
| Income(<2000Yuan/Month) | 1.15 | 0.58-2.27 | 0.686 | 1.67 | 0.69-4.03 | 0.253 | 1.18 | 0.59-2.51 | 0.662 | 2.07 | 0.97-4.38 | 0.058 | Income(>5000Yuan/Month) |
| Income(2000-5000Yuan/Month) | 1 | 0.63-1.58 | 0,982 | 1.68 | 0.99-2.83 | 0.053 | 1.1 | 0.69-1.75 | 0.701 | 0.98 | 0.58-1.64 | 0.936 | Income(>5000Yuan/Month) |
| Age at menarche^a^ | 0.98 | 0.81-2.12 | 0.435 | 1.12 | 0.96-1.30 | 0.155 | 0.92 | 0.80-1.04 | 0.182 | 0.91 | 0.80-1.04 | 0.176 | * |
| Times of abortion(N=0)^a^ | 0.84 | 0.54-1.33 | 0.458 | 0.99 | 0.56-1.78 | 0.984 | 0.93 | 0.56-1.53 | 0.76 | 1.01 | 0.60-1.69 | 0.973 | Times of abortion(1≤N≤2) |
| Times of abortion(N≥3)^a^ | 1.39 | 0.87-2.20 | 0.166 | 1.01 | 0.57-1.78 | 0.984 | 1.34 | 0.83-2.17 | 0.237 | 1.09 | 0.64-1.85 | 0.764 | Times of abortion(1≤N≤2) |
| Parity(0)^a^ | 1.29 | 0.60-2.81 | 0.515 | 0.86 | 0.37-2.05 | 0.739 | 1.47 | 0.67-3.21 | 0.337 | 2.42 | 1.23-4.76 | 0.011 | Parity(N=1) |
| Parity(≥2)^a^ | 0.83 | 0.46-1.51 | 0.545 | 0.75 | 0.32-1.78 | 0.514 | 0.53 | 0.30-0.96 | 0.036 | 0.77 | 0.42-1.41 | 0.393 | Parity(N=1) |
| mKMI>14 | 7.81 | 5.17-11.80 | <0.001 | 10 | 5.83-17.17 | <0.001 | 5.34 | 3.53-8.09 | <0.001 | 6.33 | 4.08-0.82 | <0.001 | mKMI≤14 |
| E2 T1 | 0.97 | 1.06-1.73 | 0.873 | 1.24 | 0.72-2.13 | 0.434 | 0.75 | 0.49-1.15 | 0.192 | 0.68 | 0.44-1.06 | 0.088 | E2 T3 |
| E2 T2 | 1.09 | 1.07-1.77 | 0.805 | 1.43 | 0.52-3.93 | 0.492 | 1.41 | 0.44-2.95 | 0.785 | 0.85 | 0.37-1.99 | 0.714 | E2 T3 |
| P Q1 | 0.89 | 0.50-1.60 | 0.704 | 0.92 | 0.56-1.74 | 0.626 | 0.48 | 0.48-0.26 | 0.022 | 0.82 | 0.38-1.74 | 0.594 | P Q4 |
| P Q2 | 0.82 | 0.46-1.46 | 0.501 | 0.83 | 0.49-1.67 | 0.136 | 0.82 | 0.44-1.53 | 0.531 | 0.78 | 0.38-1.60 | 0.494 | P Q4 |
| P Q3 | 1.31 | 0.73-2.35 | 0.368 | 0.99 | 0.34-2.16 | 0.834 | 0.49 | 0.27-0.95 | 0.033 | 0.69 | 0.34-1.38 | 0.295 | P Q4 |
| FSH Q4 | 1.31 | 0.78-2.19 | 0.303 | 0.88 | 0.46-1.70 | 0.705 | 1.85 | 1.04-3.28 | 0.036 | 1.71 | 0.93-3.11 | 0.082 | FSH Q1 |
| FSH Q3 | 1.21 | 0.71-2.05 | 0.48 | 1.25 | 0.67-2.33 | 0.475 | 1.77 | 0.97-3.24 | 0.064 | 1.5 | 0.83-2.69 | 0.178 | FSH Q1 |
| FSH Q2 | 0.95 | 0.55-1.62 | 0.846 | 0.64 | 0.33-1.21 | 0.17 | 1.81 | 1.02-3.23 | 0.043 | 1.27 | 0.73-2.23 | 0.399 | FSH Q1 |
| LH Q4 | 1.11 | 0.65-1.90 | 0.695 | 0.86 | 0.44-1.68 | 0.659 | 2.08 | 1.16-3.73 | 0.014 | 1.22 | 0.68-2.18 | 0.506 | LH Q1 |
| LH Q3 | 0.91 | 0.55-1.52 | 0.721 | 0.99 | 0.51-1.93 | 0.976 | 1.83 | 1.02-3.29 | 0.043 | 1.59 | 0.89-2.86 | 0.119 | LH Q1 |
| LH Q2 | 0.87 | 0.52-1.45 | 0.59 | 0.78 | 0.42-1.46 | 0.441 | 2.17 | 1.18-4.01 | 0.013 | 0.98 | 0.54-1.78 | 0.94 | LH Q1 |
| T Q1 | 0.64 | 0.34-1.20 | 0.163 | 1.75 | 0.82-3.74 | 0.148 | 0.85 | 0.45-1.62 | 0.628 | 0.74 | 0.37-1.49 | 0.393 | T Q4 |
| T Q2 | 0.87 | 0.45-1.69 | 0.687 | 1.25 | 0.59-2.68 | 0.564 | 1.12 | 0.58-2.14 | 0.742 | 0.89 | 0.41-1.92 | 0.757 | T Q4 |
| T Q3 | 1 | 0.51-1.97 | 0.994 | 1.75 | 0.83-2.89 | 0.113 | 0.77 | 0.41-1.46 | 0.422 | 0.96 | 0.46-1.98 | 0.901 | T Q4 |

Values are presented as OR (95% CI).Adjusted for Age, place of residence, level of education, employment, income, parity, times of abortion, age at menarche and BMI.

^a^ Values are presented as OR (95% CI).Adjusted for Age, place of residence, level of education, employment status, income, parity, times of abortion, age at menarche and BMI.

CI=confidence interval, OR=odds ratio.

*variables was analysis as continuous variable
